# Relevance of novel inflammatory markers in stroke-induced immunosuppression

**DOI:** 10.1186/1471-2377-14-41

**Published:** 2014-03-06

**Authors:** András Folyovich, Enikő Biró, Csaba Orbán, Anna Bajnok, Viktória Varga, Anna K Béres-Molnár, Barna Vásárhelyi, Gergely Toldi

**Affiliations:** 1 Department of Neurology, Szent János Hospital, Budapest, Diós árok 1–3 H-1125, Hungary; 2 First Department of Pediatrics, Semmelweis University, Budapest, Bókay u. 53–54 H-1083, Hungary; 3 Department of Laboratory Medicine, Semmelweis University, Budapest, Nagyvárad tér 4 H-1089, Hungary

**Keywords:** CD4+ lymphocyte, CD64+ neutrophil, CRP, Stroke, suPAR, Treg

## Abstract

**Background:**

Acute ischemic stroke (AIS) has a biphasic effect on the peripheral immune system. The initial inflammatory response is followed by systemic immunosuppression, referred to as stroke-induced immunosuppression (SIIS), leading to severe complications in stroke patients. We aimed to identify an inflammatory marker that best represents this biphasic immunological response after AIS.

**Methods:**

We investigated the alteration of CRP, WBC, neutrophil count, suPAR levels, CD4+ CD25high Tregs, CD64+ and CD177+ neutrophils and monocytes in 12 acute ischemic stroke patients free of infection within 6 hours and one week after the insult. As controls, 14 age-matched healthy individuals were included.

**Results:**

CRP, WBC and neutrophil count values were comparable in stroke patients within 6 hours and controls, however, they were elevated in stroke one week after the insult. suPAR levels were higher in both stroke groups compared to controls. The prevalence of CD64+ neutrophils was higher in stroke patients within 6 hours than in controls and it decreased in stroke one week after the insult below the level in controls (5.95 [5.41-8.75] % vs. 32.38 [9.21-43.93] % vs. 4.06 [1.73-6.77] %, p < 0.05).

**Conclusions:**

Our pilot study identified that the prevalence of CD64+ neutrophils may reflect a biphasic alteration of the immune response following AIS. Since its level decreases below baseline after one week of the CNS insult in stroke patients without infection, it might serve as a reliable candidate to identify the developing inflammatory response due to infection after stroke in the future.

## Background

Animal data clearly support a biphasic effect of stroke on the peripheral immune system. The initial phase is characterized by a local and then a generalized inflammatory response, resulting in a massive production of inflammatory factors and extravasation of lymphocytes to the brain. This early activation phase is followed by systemic immunosuppression, referred to as stroke-induced immunosuppression (SIIS) that is manifested within days of focal stroke by a reduction in T cell activation and a profound loss of T and B cells [1]. While both immune activation and suppression can be observed in stroke patients and in experimental stroke, the temporal relationship of these immune alterations has not been clearly delineated.

The role of aberrant systemic immune function in post-stroke infection has only been recently demonstrated in clinical studies [2]. Infection during the first days after AIS occurs in 25-65% of patients [3]. Pneumonia and urinary tract infection are the most common infectious complications [4], causing a significant increase in the duration of hospitalization. Therefore, from a clinical point of view, it is of great importance to distinct the inflammatory response induced by CNS damage and that later caused by evolving infection. Markers of infection currently used in clinical practice are unreliable for the distinction between the two different origins of an inflammatory response in AIS. Stroke is associated with an increase of classic markers of the inflammatory response, such as C-reactive protein (CRP) [5,6], erythrocyte sedimentation rate (ESR) [6], total peripheral white blood cell (WBC) count [7], peripheral neutrophil count [6] and body temperature [8]. It has also been demonstrated though that the elevation of the above markers is independent of the presence of infection [9]. Nevertheless, novel markers of inflammation have been emerging and becoming available for clinical diagnostic testing. Among these markers the two most promising ones are soluble urokinase plasminogen activator receptor (suPAR) and the CD64 antigen expression.

suPAR is a biomarker increasingly used for the monitoring of systemic inflammation. suPAR is derived from the cleavage and release of the membrane-bound protein, urokinase-type plasminogen activator receptor (uPAR), expressed by various cell types, such as trophoblasts, endothelial cells, smooth muscle cells, certain tumor cells, and most importantly, immunologically active cells including monocytes, activated T lymphocytes and macrophages [10]. CD177, also known as NB1 is a 55-kDa receptor that belongs to the uPAR family. It is expressed on a subpopulation of neutrophils and monocytes, and is regarded as a marker of extravasation [11]. Cell migration is tightly linked to adhesion and chemotaxis. The uPAR system is directly involved in these mechanisms [12]. suPAR is detectable in low, but constant concentrations in plasma of healthy individuals. However, activation of the immune system and the development of an inflammatory response lead to elevated plasma suPAR concentrations [13].

In resting neutrophils, Fc gamma receptor I (FcgRI, CD64) is expressed at very low levels; upon neutrophil activation it is strongly upregulated by the proinflammatory cytokines IFN-gamma and granulocyte colony stimulating factor (G-CSF) which are produced during infections or exposure to endotoxin [14]. Monocytes also express CD64 and upregulate this receptor during activation. Neutrophil and monocyte CD64 expression, demonstrated using flow cytometry, can be used as a diagnostic marker of infection and sepsis. Neutrophil CD64 is superior to CRP and hematological determinations for detecting systemic infection or sepsis, since it combines high sensitivity (above 90%) with high specificity (90-100%) [15].

In this study, we aimed to describe the alteration of selected clinical inflammatory markers (CRP, WBC, neutrophil count, suPAR, CD64 neutrophil and monocyte antigen expression) in AIS in order to identify an inflammatory marker that best represents the biphasic immunological response following the CNS insult. We also aimed to describe the prevalence of immunosuppressive CD4+ CD25high Treg cells, activated (CD11b+) monocytes, as well as the prevalence of CD177+ neutrophils and monocytes.

## Methods

Peripheral blood samples were taken from 12 identical AIS patients within 6 hours (Stroke 1) and one week after the insult (Stroke 2). AIS was defined according to the WHO definition [16]. Patients with subarachnoid haemorrhage, epidural or subdural hemorrhage, transient ischaemic attack (TIA) or neurological deficit due to trauma or neoplasm were excluded. Based on positive microbial cultures, patients with infection after stroke were also excluded. The severity of stroke was assessed using the modified NIHSS and Rankin scores upon admission [17]. As controls, 14 age-matched healthy individuals with similar cardiometabolic risk factors were included from whom peripheral blood samples were taken on a single occasion. Healthy controls had a negative history of stroke or other neurological disorders. Demographic data and risk factor profiles of patients and controls are shown in Table 1. Written informed consent was obtained from all subjects, and our study was reviewed and approved by an independent ethical committee of the institution (Szent János Hospital, Budapest). Laboratory studies and interpretations were performed on coded samples lacking personal and diagnostic identifiers. The study was adhered to the tenets of the most recent revision of the Declaration of Helsinki.

**Table 1:** Demographic data and risk factors of controls and acute ischemic stroke patients

	**Control (n = 14)**	**Stroke (n = 12)**
Age, years	62,5 [52–71]	67,5 [60–76]
Male/female	7/7	4/8
Hypertension	7 (50%)	9 (75%)
Smoking	3 (21%)	4 (33%)
Alcohol	4 (29%)	4 (33%)
Obesity	4 (29%)	4 (33%)
Earlier CVD	4 (29%)	5 (42%)
Cholesterinemia	2 (14%)	2 (17%)
Diabetes	2 (14%)	4 (33%)
Atrial fibrillation	2 (14%)	4 (33%)
Modified rankin score	-	4 (1–5)
NIHSS score at admission	-	5.5 [4-15]
TOAST classification	-	1: 5 (42%); 2: 4 (33%); 3: 3 (25%)
Largest diameter of the infarct, mm	-	27 [16–37,5]

Data are expressed as median [IQR] or number (%). CVD – cardiovascular disease.

**Figure 1:**
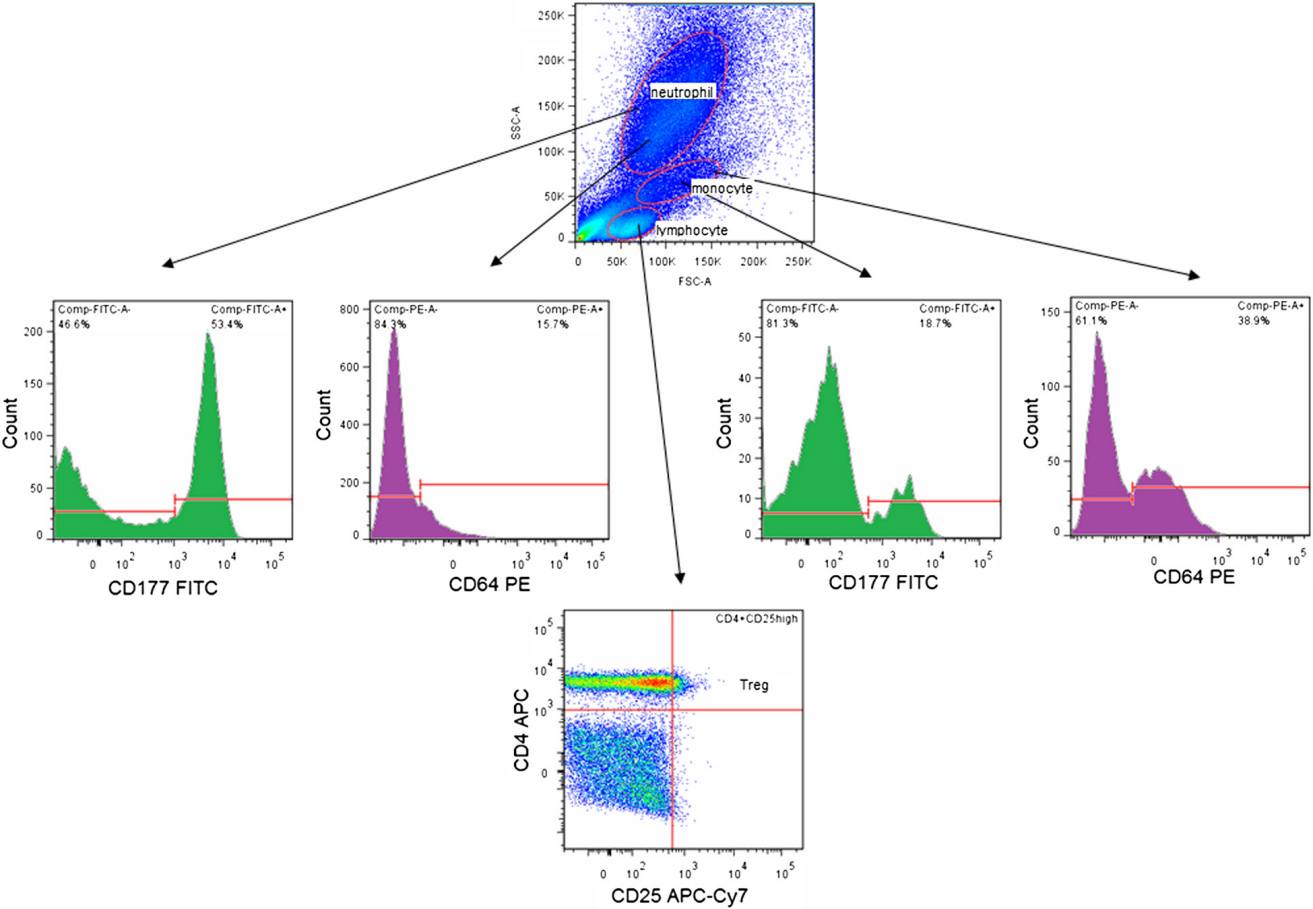
**Gating strategy for flow cytometry measurements.** FSC – forward scatter, SSC – side scatter, Treg – regulatory T cells.

Plasma was isolated from heparin anticoagulated blood samples and stored at −80°C until measurement. Plasma suPAR concentrations were measured with the suPARnostic Flex ELISA assay (ViroGates A/S, Birkerød, Denmark). CRP levels were measured using a Roche Hitachi 912 instrument with Roche Tina-quant CRP immuno-turbidimetric assay (Roche Diagnostics GmbH, Mannheim, Germany). Blood count values were determined using a Beckman Coulter UniCel DxH800 analyzer (Beckman Coulter, Brea, CA, USA).300 ul heparin anticoagulated whole blood was incubated with the following conjugated anti-human antibodies: CD4 APC (Miltenyi Biotec, Bergisch Gladbach, Germany), CD11b PE-Cy7 (BD Biosciences, San Jose, CA, USA), CD25 APC-Cy7 (BioLegend, San Diego, CA, USA), CD177 FITC (BioLegend), CD64 PE (BD Biosciences). After 30 minutes of incubation at 4°C, we added FACS Lysing Solution to each sample (BD Biosciences). Cells were incubated for 20 min in dark at room temperature and then were washed. After washing, 500,000 cells were recorded from each sample. Flow cytometry measurements were conducted on a BD FACSAria flow cytometer (BD Biosciences). Data acquired from the measurements were evaluated with FlowJo software (version 7.6.3, Tree Star, Ashland, OR, USA). The gating strategy is shown in Figure 1.

Data are expressed as median [interquartile range]. CRP values below the level of detection (1 mg/L) were regarded as 1 mg/L. Comparisons between healthy individuals and AIS patients were made with Mann–Whitney tests. In case of comparisons between samples taken at different time points of AIS patients, the Wilcoxon signed rank test was performed. Correlation analyses were performed using Spearman’s tests. p values less than 0.05 were considered significant. Statistics were calculated using the SPSS software (version 20.0, SPSS, Inc. Chicago, IL, USA).

## Results

**Figure 2:**
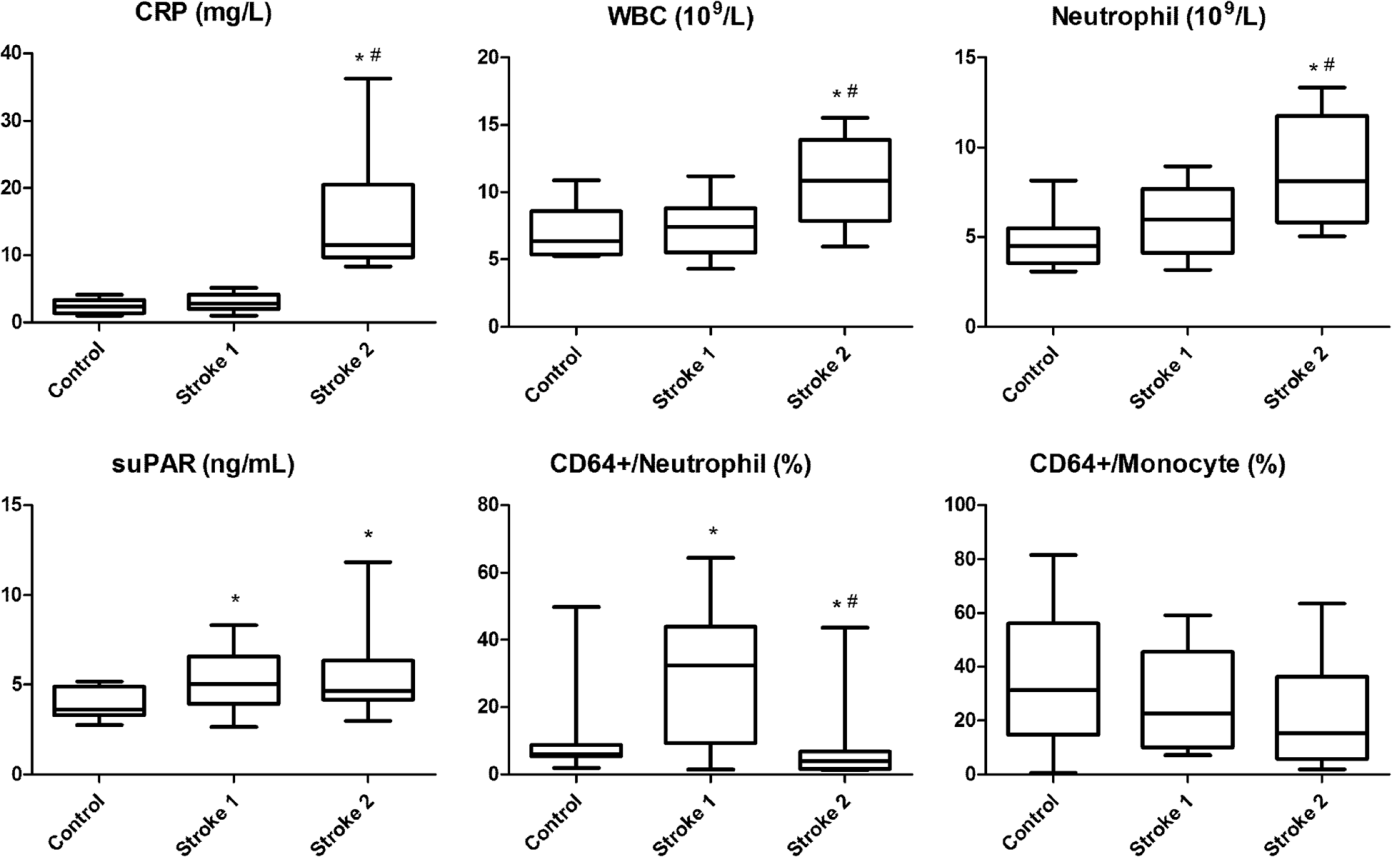
**Selected inflammatory parameters in controls and acute ischemic stroke patients measured within 6 hours (Stroke 1) and one week after (Stroke 2) the insult.** The prevalence of CD64+ neutrophils may reflect a biphasic alteration of the immune response in acute ischemic stroke patients free of infection (inflammation followed by immunosuppression). Horizontal line – median, box – IQR, whisker – range. *p < 0.05 vs. Control, ^#^p < 0.05 vs. Stroke 1. CRP – C-reactive protein, suPAR – soluble urokinase plasminogen activator receptor, WBC – white blood cell count.

Our results are summarized in Figure 2 and Table 2.

**Table 2:** Inflammatory parameters in controls and acute ischemic stroke patients measured within 6 hours (stroke 1) and one week after (stroke 2) the insult

	**Control (n = 14)**	**Stroke 1 (n = 12)**	**Stroke 2 (n = 12)**
suPAR, ng/mL	3.62 [3.30-4.91]	5.04 [3.93-6.58]^a^	4.64 [4.15-6.35]^a^
CRP, mg/L	2.37 [1.40-3.36]	2.83 [1.98-4.15]	11.48 [9.73-20.52]^ab^
WBC, 10^9^/L	6.38 [5.35-8.58]	7.42 [5.50-8.81]	10.87 [7.85-13.90]^ab^
Neutrophil, 10^9^/L	4.50 [3.54-5.49]	5.97 [4.11-7.68]	8.10 [5.82-11.75]^ab^
CD64+/neutrophil, %	5.95 [5.41-8.75]	32.38 [9.21-43.93]^a^	4.06 [1.73-6.77]^ab^
CD64 MFI/neutrophil, au	262 [145–505]	331 [205–528]	258 [93–838]
CD177+/neutrophil, %	53.24 [44.10-65.58]	76.95 [47.18-89.68]^a^	73.46 [44.55-84.28]^a^
CD177 MFI/neutrophil, au	3780 [2112–4859]	5887 [4429–9455]	6208 [3143–6899]
CD64+ CD177+/neutrophil, %	2.78 [1.71-3.79]	21.30 [0.98-31.58]	0.99 [0.80-1.79]^ab^
Monocyte, 10^9^/L	0.40 [0.38-0.53]	0.45 [0.33-0.68]	0.50 [0.40-0.68]
CD11b+/monocyte, %	83.50 [68.30-89.03]	84.55 [73.60-91.22]	89.90 [76.08-94.03]
CD64+/monocyte, %	31.32 [14.65-56.20]	22.51 [10.00-45.65]	15.29 [5.80-36.34]
CD64 MFI/monocyte, au	280 [69–610]	955 [223–1658]	142 [42–365]^b^
CD177+/monocyte, %	26.09 [11.81-40.53]	36.15 [23.83-61.22]^a^	43.47 [30.52-67.97]^a^
CD177 MFI/monocyte, au	2293 [1378–3625]	4705 [1774–6483]	2492 [1479–3503]
CD64+ CD177+/monocyte, %	1.43 [0.65-2.36]	2.50 [0.69-4.21]	1.15 [0.77-1.94]
Lymphocyte, 10^9^/L	1.49 [0.96-2.25]	1.24 [0.70-1.50]	1.34 [0.53-1.84]
CD4+/lymphocyte, %	35.41 [27.51-44.17]	9.08 [3.85-23.96]^a^	22.13 [18.41-41.86]^b^
CD4+ CD25high/CD4+, %	9.38 [7.52-13.95]	10.46 [9.40-13.13]	14.59 [8.97-18.25]

Data are expressed as median [IQR]. ^a^p < 0.05 vs. Control, ^b^p < 0.05 vs. Stroke 1. au – arbitrary unit, CRP – C-reactive protein, MFI – mean fluorescence intensity, suPAR – soluble urokinase plasminogen activator receptor, WBC – white blood cell count.

CRP, WBC and neutrophil count values were comparable in Stroke 1 and controls, however, they were elevated in Stroke 2. No difference in monocyte and lymphocyte counts or the prevalence of CD11b + monocytes was observed between the study groups. The prevalence of CD4+ lymphocytes decreased in Stroke 1 compared to controls, and increased again in Stroke 2 compared to Stroke 1. The prevalence of CD4+ CD25high Treg cells did not differ significantly between the study groups, although a tendency of increased Treg prevalence was observed in Stroke 2.

suPAR levels were higher in stroke patients at both time points compared to controls.

The prevalence of CD64+ neutrophils was higher in Stroke 1 than in controls. However, their prevalence decreased in Stroke 2 below the level in controls. The prevalence of CD177+ neutrophils was higher in both stroke groups compared to controls. Mean fluorescence intensity (MFI) of CD64 and CD177 in neutrophils was comparable in all study groups. The prevalence of CD64+ CD177+ neutrophils was lower in Stroke 2 compared to the two other study groups, and showed a tendency of elevation in Stroke 1 compared to controls.

The prevalence of CD64+ monocytes was comparable in all study groups, while that of CD177+ monocytes was higher in both stroke groups compared to controls. MFI of CD64 in monocytes was lower in Stroke 2 compared to Stroke 1, while that of CD177 was comparable in all study groups. The prevalence of CD64+ CD177+ monocytes was also comparable in all study groups.

No correlation was detected between plasma suPAR levels and the expression of CD177 on neutrophils or monocytes in any of the study groups. Furthermore, we found no correlation between markers of stroke severity (modified Rankin and NIHSS scores) and the prevalence of CD64+ neutrophils in AIS patients.

## Discussion

Stroke is a major cause of disability and mortality in developed countries [18]. However, this is not only due to the neurological deficit caused by the brain lesion. It is now clear that AIS results in multi-organ systemic disease that occurs within the context of the complex interplay between the brain and immune system. While acute stroke patients may survive the initial CNS insult, subsequent complications might develop over time. SIIS, resulting in an increased risk of infections, is the most common of these complications, causing a significant increase in the duration of hospitalization [19]. SIIS also hinders the regeneration process of the CNS, and therefore worsens the functional outcome of patients. Therefore, in our pilot study we aimed to describe the alteration of selected clinical inflammatory markers in AIS.

As outlined below, most investigations report that alterations caused by SIIS are already present one week following the insult. Therefore, we chose to analyze patient samples one week after admission. Compared to our findings, Emsley et al. reported that CRP concentration was significantly higher in AIS patients earlier, even at admission, and remained elevated until 3 months in comparison with control subjects, reaching a peak at 5–7 days after stroke onset. Similarly, total WBC and neutrophil counts were elevated from admission until 3 months. By 12 months, no differences from control subjects were detectable in CRP, WBC or neutrophil values. Similar differences in these values were seen when patients with evidence of infection were excluded, although they were somewhat less marked [9]. According to the authors, the relatively early increase in these markers after stroke may have two possible explanations. First, that stroke induces a very early inflammatory response that is sustained for a long time. Second, the data may indicate a pre-existing inflammatory condition in stroke patients which could contribute to the development of stroke. The concept of a pre-existing inflammatory condition is supported by evidence that low-grade inflammation, identified by an elevated CRP concentration, may be a risk factor for stroke [20]. However, this concept is not supported by our data, indicating no difference between CRP values of the Stroke 1 group and controls. In the report of Emsley and colleagues, median admission CRP for all patients was raised relative to controls, although this increase was not significant for patients sampled less than 4 hours from onset [9]. Therefore, the difference between their and our findings at admission in these values might be due to different chronologic definition of the first blood sampling.

It has recently gained attention that cerebral ischemia does not only lead to local inflammation of the brain tissue and a generalized inflammatory response, but also to a dramatic loss of peripheral blood T cells with subsequent infections. Therefore, as well as evidence of immune activation, there is also evidence of post-stroke immune suppression. The results of Vogelgesang et al. indicated that stroke induced a dramatic and immediate loss of T lymphocytes, most pronounced within 12 hours after stroke onset. Only patients with subsequent infection exhibited a delay in the recovery of CD4+ T lymphocyte counts. They suggest that a loss of CD4+ T cells contributes to SIIS. The authors argue that CD4+ T cell count on the day after stroke may emerge as a predictive marker for post-stroke infection [21]. Their observation is reinforced by our findings, indeed showing a loss of CD4+ T cells even within 6 hours after stroke onset, and a recovery of CD4+ T cell prevalence one week after the insult in the lack of infection. While both immune activation and immune suppression can be observed in stroke patients and in experimental stroke, the temporal relationship of these immune alterations has not been clearly delineated. Also in the population studied here the temporal alterations in immune functions may be more complex.

Yan et al. suggested that SIIS is also likely to be due to increased immunoregulatory activity, possibly by activation of Treg cells. Indeed, they found that the percentages of FoxP3+ regulatory T cells were significantly increased from day 7 following the CNS insult in patients with AIS compared to healthy subjects [22]. Although the prevalence of CD4+ CD25high Treg cells did not differ significantly between the study groups in our investigation, a tendency of increased Treg prevalence was observed one week after the insult compared to the initial values in stroke patients and controls. The difference seen between our and their results might be due to the fact that different markers were used for the detection of Tregs (in our study, this subset was described as CD4+ CD25high cells, and FoxP3 was not applied) and that the severity of stroke was different in the two investigations. The patients examined by Yan et al. had relatively mild disease (average NIHSS was 3.6, corresponding to minor stroke), while our patients developed severe disability already within 6 hours (average modified Rankin score was 4).

suPAR has earlier been demonstrated to be elevated in sera, but not in CSF of stroke patients [23]. Our results indicate that suPAR levels were elevated already within 6 hours of the insult, earlier than CRP, WBC or neutrophil counts. Hence, suPAR is an earlier indicator of the inflammatory response developing due to the CNS insult. The increase in the prevalence of uPAR expressing (CD177+) neutrophils and monocytes indicates the higher susceptibility of these cells for extravasation and infiltration of the lesion both within 6 hours and one week after the insult. MFI values of CD177 were not altered, showing that the amount of receptors per cell is constant. Interestingly, we could not detect a correlation between plasma suPAR levels and the expression of CD177 on neutrophils or monocytes in any of the study groups. suPAR is believed to have inhibiting properties on uPAR-dependent adhesion, as it competes with cell surface bound uPAR for binding integrin and vitronectin molecules at the focal contacts [24]. This competition might have a regulatory effect on uPAR expression and on the cleavage of the membrane-bound form of the protein.

Neutrophil granulocytes express FcgRI (CD64) antigen only when activated. Its expression rises in bacterial infection, thus it is increasingly used as a diagnostic marker for sepsis [15]. Based on our current findings, influences other than infection, such as stroke-related inflammation may also rapidly increase CD64 expression. Our results indicate that the prevalence of CD64+ neutrophils increase within 6 hours and falls back by one week after the CNS insult even below the baseline measured in the healthy control group, most closely representing the biphasic immunological response characteristic for AIS (inflammation followed by immunosuppression). Therefore, upon infection, the prevalence of CD64+ neutrophils may rise again, while since the level of the other investigated inflammatory parameters remain elevated even without infection, their alteration would not be specific for inflammation of infectious origin. The decrease of CD64 expression on neutrophils might be due to the evolving immuno-suppressive mechanisms and the consolidation of the inflammatory response. In contrast to neutrophils, such alterations were not detected in the prevalence of CD64+ monocytes, although MFI of the CD64 antibody did decrease one week after the insult compared to the onset of stroke.

Limitations of our study include small sample size, differences in sex ratio of patients and controls and the lack of AIS patients with confirmed infection. Based on our current results, more focused studies in the future may also provide an opportunity to perform detailed analysis on subgroups of stroke patients. Further subgroup analysis based on stroke subtype on larger samples might also provide additional information on differences of the degree of inflammatory activation. For instance, Licata et al. observed significant differences in inflammatory parameters between lacunar and non-lacunar ischemic stroke patients [25-27].

## Conclusions

In conclusion, our pilot study identified that the prevalence of CD64+ neutrophils may reflect a biphasic alteration of the immune response following AIS. Since its level decreases below baseline after one week of the CNS insult in AIS patients without infection, it might serve as a reliable candidate to identify the developing inflammatory response due to infection after stroke. However, further clinical studies are needed to test its usefulness in the distinction of the infection-induced inflammatory response in AIS patients developing infection.

## Competing interests

The authors declare that they have no competing interests.

## Authors’ contributions

AF participated in sample collection and interpretation of results and drafted manuscript, EB performed measurements and drafted manuscript, CO performed measurements and analyzed data, AB performed measurements and analyzed data, VV participated in sample collection and interpretation of results, AKB-M participated in sample collection and designed study, BV designed study, participated in interpretation of results and critically read manuscript, TG designed study, participated in interpretation of results and drafted manuscript. All authors read and approved the final manuscript.

## Pre-publication history

The pre-publication history for this paper can be accessed here:

http://www.biomedcentral.com/1471-2377/14/41/prepub
